# Recent records of steppe species in Belarus, first indications of a steppe species invasion?

**DOI:** 10.3897/zookeys.100.1541

**Published:** 2011-05-20

**Authors:** Oleg Aleksandrowicz

**Affiliations:** Pomeranian University, Slupsk, Poland

**Keywords:** Carabidae, Belarus, steppe species, geographic ranges

## Abstract

Belarus is situated at a crossroad of natural borders of species distributions: the NE part is situated in a taiga zone, whereas the other part of terrain is in the European forest zone. The distance of Belarus to the steppe zone is about 330 kilometers. This geographical position and the extensive knowledge of its fauna can be used to monitor changes in the distribution of different species. An intensive study of open habitat ground beetles was carried out from 1975–2008 in Belarus, using pitfall traps, quadrate-sampling methods, hand collecting, netting and light traps. In total, more than 130 000 specimens of ground beetles belonging to 169 species were collected from 62 fields and 11 meadows of different types. 217 specimens of *Calosoma investigator* (Illiger 1798), 2 specimens of *Calosoma denticolle* (Gebler 1833), and one specimen of *Harpalus subcylindricus* (Dejean, 1829), *Harpalus honestus* (Duftschmid 1812) and *Zabrus tenebrioides* (Goeze 1777) were present in this material. All specimens were macropterous and exclusively caught at fields and waste grounds on sandy soil. Nowadays Belarus is the northernmost location for these species in Eastern Europe. Steppe species most probably migrated to SE Belarus from NE Ukraine, using Dnieper and its river valleys. The shift in the geographic distribution of steppe species during the last thirty years in Belarus have been attributed to a higher frequency of warmer and wetter summers in the last few decades.

## Introduction

Every aspect of an insect’s life cycle depends on temperature. As such, these organisms respond quickly to climatic changes by shifting their geographical distribution. This quick response allows them to take advantage of new climatic environments. A wide variety of vertebrate and invertebrate species have moved northwards and uphill in response to global warming. These changes have already been documented in Europe (Ohlemuller et al. 2006).

Similar shifts in geographic distribution were also documented among well-studied insect groups in Belarus (Eastern Europe). In the last decade, xerophilous steppe species from different insect orders were recorded from SE Belarus, for example: *Scolia hirta* (Schrank 1781) and *Megascolia maculata* (Drury 1773) (Prischepchik 2008), *Mantis religiosa* (Linnaeus 1758) (Dictyoptera, Mantidae) (Kulak 2009), and *Zerynthia polyxena* (Denis et Schiffermuller 1775) (Lepidoptera, Papilionidae) (Krasnaya Kniga Respubliki Belarus).

**Figure 1.**
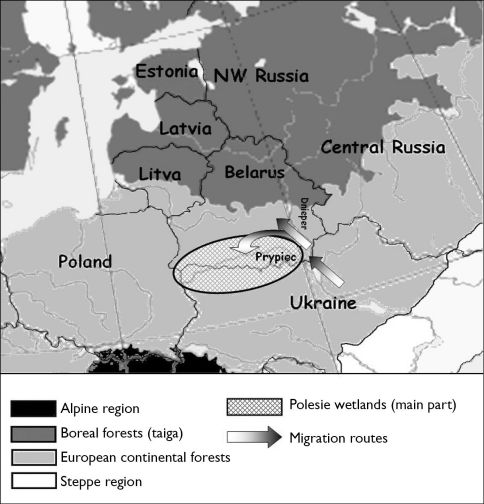
Biogeographic regions and the geographic location of Belarus.

Belarus is situated at a crossroad of natural borders of species distributions: according to the biogeographical regionalization of Europe (Biogeographical provinces) the northeastern part is situated in the taiga zone, whereas the rest of the terrain is within the European forest zone (Fig. 1). The distance from Belarus to the steppe zone is about 330 kilometers.

This geographic position makes Belarus ideal for monitoring changes in the geographic distribution of the fauna. Fortunately, the fauna of South East Belarus has been studied extensively, which is well documented in publications and collection material.

At the end of the 19th century the beetles of South East Belarus were one of the most intensively studied groups within the Russian Empire. The first checklist of Mogilev province (including the Mogilev and Gomel regions of Belarus) was compiled by Arnold (1901). In 1992 a check was made of the list of carabid beetles as found by Arnold. This list includes 120 species is housed in the Museum of the Zoological Institute of the Academy of Science of Russia in St. Petersburg (Aleksandrowicz 1992).

The faunistic inventory of carabids was continued in the second half of 20th century (Gorbunova 1956; Aleksandrowicz 1979, 1991). In the last 20 years an intensive study in South East Belarus was executed to analysis of the distribution, abundance and occurrence of carabid beetles. The aim of this study is to determine whether changes occurred in the carabid fauna of Belarus.

## Location, methods and material

Belarus is situated in eastern Europe, on the eastern of Poland (53°00'N, 28°00'E). The total territory of Belarus is 207.6 thousand km2 (Fig. 1. Landscape and climate).

Climate in Belarus is moderately continental: a transitional form of maritime to continental climate with mild and humid winters, warm summers and damp autumns.

The terrain is generally flat and contains much marshland, especially in the southern part near the Ukraine border, which is named Polesie (Fig 1. Wetlands). The Polesie lowlands lie mainly along the Pripyat river and occupy 80 000 km2. The Polesie area presents a plain with rare and irregularly distributed hills with flat tops and gentle slopes. Sand is common in places of higher elevation on which pine trees are typical. Wide and swampy river valleys are a characteristic feature of the landscape. The Polesie lowlands are only 100–150 m a.s.l., with the western part slightly higher than the eastern parn. During the last 20 years, intensive drainage of the Polesie swamps has occurred. The aim of this activity is to turn the swamps into hay-fields. Many swamps have disappeared, and many canals have been cut through the region (Belarusian Lowlands).

Intensive inventories of open habitat carabids in 1975–1976 were carried out in Belarus Polesie, using pitfall traps and quadrate sampling method (0.25 m2). These studies have been repeated in the area of Luninetz, in the Brest district of Belarus (52°14'26"N, 26°37"E) in 1982–1983.

The entire terrain of Belarus was studied in 1980–1985 during 12 expeditions of the Belarus Institute of Plant Protection. The purpose of these expeditions was to reveal grain crop pests. Hand collecting and netting were used to collect the insects.

Studies were performed by collecting material from light traps in crop fields and orchards of the Gomel Regional Crop Protection Service in 1980–1990.

Later studies (1990-2005) evaluated the effects of insecticide and herbicide spraying on carabid communities in wheat and barley fields in the Minsk and Mogilev districts, using pitfall traps (Central and East Belarus).

In 2005–2008, faunistic inventories were continued in the east of Polesie: in the vicinity of Gomel and Polesie Radio-Ecological Reserve, using pitfall traps, netting and hand collection. The main focus was arable fields and the terrain of the Prypiatski National Park (Aleksandrowicz et al. 1996, 1997; Aleksandrowicz and Kapcjugh 2002). Also, wing development of all specimens was determined.

The level of faunistic knowledge in Belarus allows us to monitor the appearance or disappearance of carabid species. In all likelihood, steppe species will have colonised Belarus during decent decades. A steppe species is defined as a species of euroasiatic subboreal geographic ranges. These species only colonise on open (mainly arable) habitats with mostly continental climate. This definition is similar to Tischler’s (1965) definition of steppe species.

## Results

In total, during the period 1975–2008, more than 130 000 specimens of ground beetles belonging to 169 species were collected, mainly by pitfall traps from 62 fields and 11 meadows of different types.

Among this material, 217 specimens of *Calosoma investigator* (Illiger 1798) and 2 specimens of *Calosoma denticolle* (Gebler 1833) were found. *Harpalus subcylindricus* (Dejean, 1829), *Harpalus honestus* (Duftschmid 1812) and *Zabrus tenebrioides* (Goeze 1777) were represented by only one specimen.

*Calosoma investigator* is widespread in the steppe zone of Eurasia, from South-East Europe to Baikal (Catalogue of Palearctic Coleoptera 2003). Its distribution in Middle Europe is not clear. According to Lindroth (1945) it is absent from Sweden. Only one old (from the 19th century) specimen of *Calosoma investigator* is known from museum material (“Oland, Mortonson, Mus. Goteborg”) without an exact locality.

The distribution of *Calosoma investigator* in North-East Poland (Ost Preussen) is unclear. Bercio and Folwaczny (1979) concluded that *Calosoma investigator* was absent from Ost Preussen and data collected by Lesniak (1964) for northeast Poland are based on misidentifications.

In SE Europe *Calosoma investigator* is known from the Ukraine, Moldova, Romania, Bulgaria and European Turkey (Catalogue of Palearctic Coleoptera 2003). The first records in Rumania are from 1991 (Nitu 1991).

**Figure 2.**
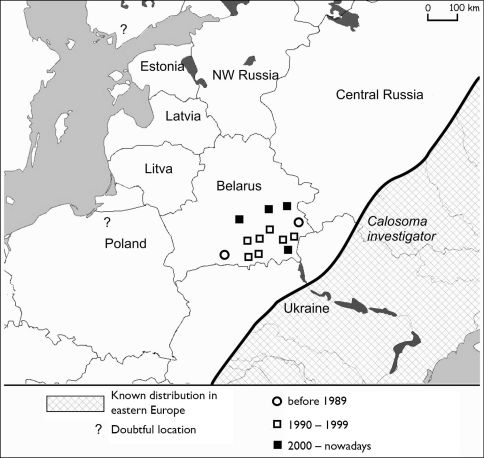
Actual distribution of *Calosoma investigator* in Belarus.

In Belarus *Calosoma investigator* was collected for the first time in 1975 near Luninetz (52°14'21"N, 26°37'46"E) in barley field. Nowadays it can be found up to Slutsk (52°57'40"N, 27°37'27"E) and Bobruysk (52°12'27"N, 29°02'25"E) (Fig. 2).

At the moment, *Calosoma investigator* occupies almost the entire southeastern part of Belarus. It inhabits arable lands on sandy soils and are sometimes locally abundant. All of the 217 collected specimens were macropterous. Their expansion rate is estimated at about 50–60 km in 10 years.

The geographic distribution of *Calosoma denticolle* is limited by the steppe zone of Eurasia, from southeast Europe to northeast China (Catalogue of Palearctic Coleoptera 2003).

In northern Europe only one specimen is known from southern Finland in an atypical locality. It was collected from the Baltic shore after a strong gale in 1935 (Lindroth 1945).

**Figure 3.**
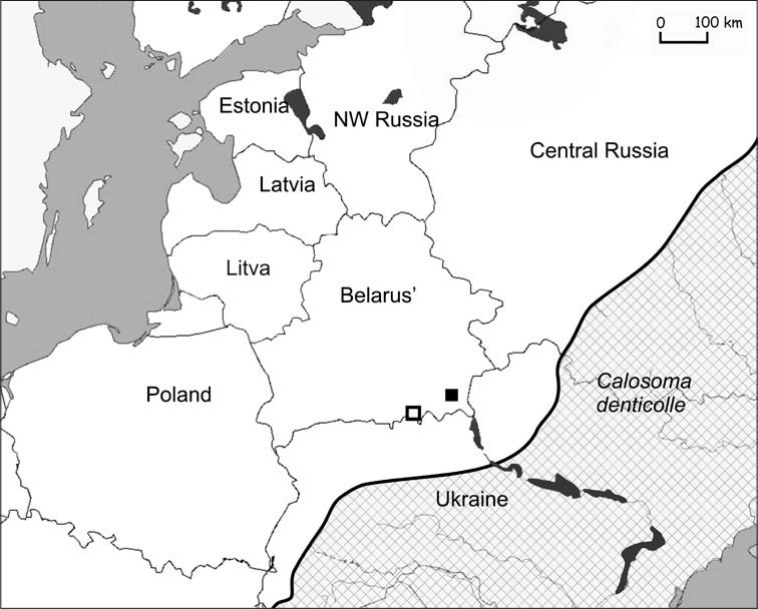
Actual catch of *Calosoma denticolle* in Belarus (□ – 1988; ■ – 2007) and its known distribution in eastern Europe (chequered area).

In Belarus it was caught for the first time in 1988 near Turov (52°3'33.29"N, 27°44'36.15"E) in an arable field. A second specimen was captured in 2007 near the village Arevithcy in a wasteland (51°36'52.72"N, 29°50'49.50E) (Fig. 3).

**Figure 4.**
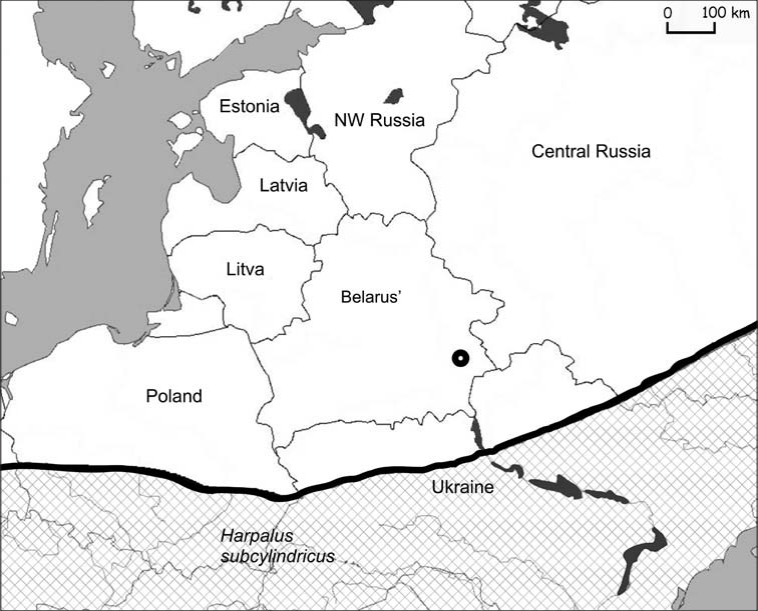
Actual catch of *Harpalus subcylindricus* in Belarus (○ – 1988) and its known distribution in eastern Europe (chequered area).

*Harpalus subcylindricus* is widespreadfrom southern Europe and southern part of Middle Europe to the Caucasus to West Asia (Catalogue of Palearctic Coleoptera 2003). It is not know from Poland, the Baltic States or northwestern Russia. Recently it has also been recorded from the southern parts of Poland (pers. comm. M. Stachowiak). In southeast Belarus one macropterous specimen was collected near Homel in a barley field in 1998 (52°22'50.85"N, 30°50'9.33"E) (Fig. 4).

**Figure 5.**
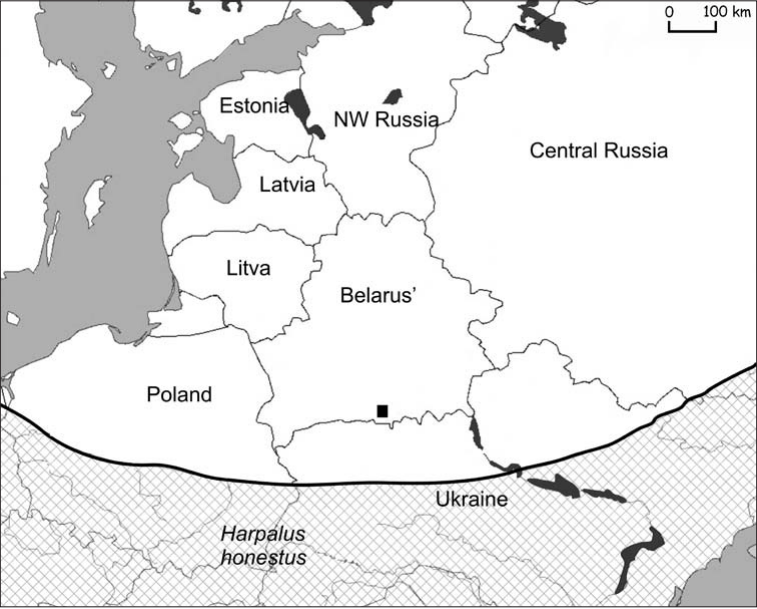
Actual catch of *Harpalus honestus* in Belarus (■ – 1997) and its known distribution in eastern Europe (chequered area).

*Harpalus* (s.str.) *honestus* is distributed from southern Europe and southern part of Middle Europe to the Caucasus to West Asia (Catalogue of Palearctic Coleoptera 2003). It is absent from the North of Poland, the Baltic States and northwestern Russia. In Belarus, one macropterous specimen was collected near the village Liaskovitchy on a sandy beach of the Pripyat river in 1997 (52° 7'3.68"N, 28°10'57.60"E) (Fig. 5).

*Zabrus tenebrioides* is widespread from southern Europe and southern part of Middle Europe and the Caucasus to Turkey. Distribution of *Zabrus tenebrioides* in the former USSR (South-West Russia, Caucasus and Ukraine) has been studied well because of its economic damage to grain crops (Interactive Agricultural Ecological Atlas). In Fennoscandia it is known from the south of Sweden and Denmark only. Old data from Latvia and Estonia are erroneous (Silfverberg 2004). So far it has never been registered from Belarus, Lithuania, northwest and north of European Russia (Kryzhanovsky et al. 1995; Aleksandrovich et al. 1996; Silfverberg 2004). As such, data from the Catalogue of Palearctic Coleoptera (2003) and the distribution map on the site Fauna Europea (Distribution of *Zabrus* (*Zabrus*) *tenebrioides*) are erroneous and should be corrected.

**Figure 6.**
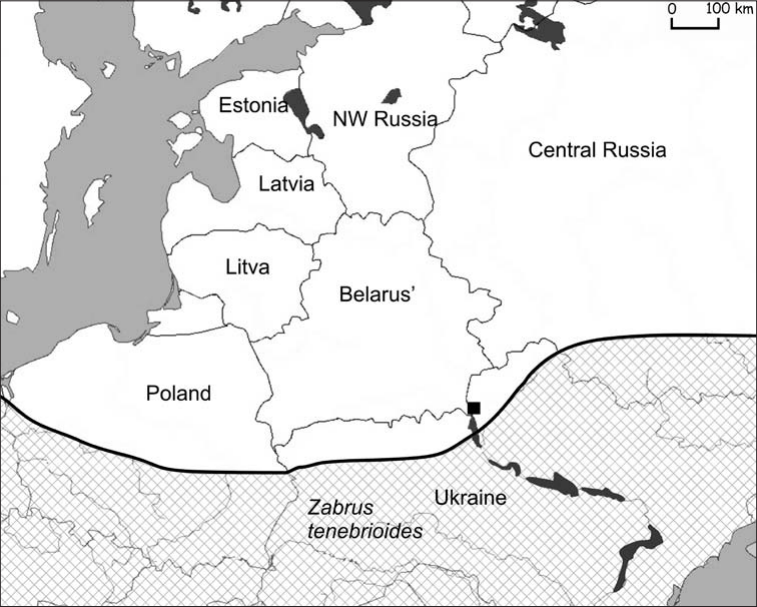
Actual catch of *Zabrus tenebrioides* in Belarus (■ – 2007) and its known distribution in eastern Europe (chequered area).

For the first time, one macropterous specimen was collected in southeastern Belarus near the village Khvashchouka, in a dry meadow in 2007 (51°38'49.18"N, 29°47'3.09"E) (Fig. 6).

## Discussion and conclusions

The data presented most likely show the northern most locations for these steppe species in eastern Europe.

The different types of forests, meadows and marshes (Gorburova 1956, Kipenvarlits 1961; Khotko et al. 1980; Aleksandrowicz 1991; Aleksandrowicz et al. 1997; Khotko 1993; Chumakou 1994), as well as anthropogenic landscapes: cities (Molodova 1990) and fields (Aleksandrowicz 1979; Molodova 1980) have been investigated by different entomologists for the last 50 years.

All steppe species found in these studies were exclusively caught in fields and waste grounds on sandy soils. This seems to confirm the theory of Elton (1958) that migrants invade arable fields or waste grounds first, where competition is lower and migrants can dispersed successfully. Of all the steppe species, only macropterous specimens have been found. Except for *Calosoma investigator* and *Calosoma denticolle* the other three steppe species were caught only as single specimens.

The new species probably migrated to the north and to the northwestern part of Belarus from the southeast (connected to the northeast part of the Ukraine). The Polesie lowland, in the south west of Belarus, harbours an extensive complex of marshes and lakes that might act as a natural barrier for migrations from northern Ukraine.

Migrations from the Ukraine probably occurred along the Dnieper river valleys and its affluent: Pripyat, Sozh and Berezina (Fig. 1). The actual distribution of steppe species in Belarus is in the valleys of these rivers (Figs 3–6). Only *Calosoma investigator* migrated beyond the river valleys and nowadays can be found on arable lands north from this river system (Fig. 2).

The changing of geographic ranges can have an economic impact on the local agricultural society. *Calosoma* species will probably be beneficial in this case as general predators. But *Zabrus tenebrioides* is known as a serious pest of winter wheat in eastern Europe and a local pest in central Europe (Kromp 1999; Mrówczyński et al. 2007).

Global or local climatic changes is frequently mentioned as one of the reasons for shifts in geographic ranges. Over a long period of observation (1881–2001) Loginov et al. (2003) estimated an average annual temperature increased of 1 °C. Average winter and spring temperatures increased even more. During this period the most significant increase in temperature took place at the end of the last and the beginning of the current century, with an average temperature increase of 3–4 °C.

This change in temperature makes it plausible that shifts in the geographic distribution of some insect species during the last thirty years in the Belarus have been the result of a higher frequency of warmer and wetter summers. To complete its life cycle, steppe species need high summer temperatures, which makes it possible for them to move further north. As in the steppe of the Ukraine and Russia, winters are colder than in Belarus (www.agroatlas.ru), and an increase of winter temperature probably does not have any impact on their distribution.

However, in the Polesie region in Belarus, intensification of agriculture and changes in land use (e.g., first of all Polesie’s peat-bog drainage) also took place. This kind of management might also cause shifts in geographic changes. These hypotheses indicate the general problem of separating climatic effects from human effects in interpreting biological patterns.
